# Editorial: Sleep disorders and cerebrovascular diseases

**DOI:** 10.3389/frsle.2025.1683754

**Published:** 2025-08-29

**Authors:** Valerio Brunetti, Eleonora Rollo, Francesca Colò, Valentina Gnoni

**Affiliations:** ^1^Unità Operativa Complessa di Neurologia, Dipartimento di Neuroscienze, Organi di Senso e Torace, Fondazione Policlinico Universitario Agostino Gemelli Istituto di ricovero e cura a carattere scientifico, Rome, Italy; ^2^Center for Neurodegenerative Diseases and the Aging Brain, University of Bari Aldo Moro at Pia Fondazione “Card. G. Panico”, Tricase, Italy; ^3^Department of Neurosciences, Università Cattolica del Sacro Cuore, Rome, Italy; ^4^Center for Experimental Neurology, Department of Neurology, Bern University Hospital (Inselspital) and University of Bern, Bern, Switzerland

**Keywords:** stroke, sleep, sleep disorders, obstructive sleep apnea, insomnia, transcranial magnetic stimulation (TMS), mild cognitive impairment, dementia

Stroke is a leading cause of mortality and long-term disability, with significant personal, societal, and economic consequences (GBDS Collaborators, 2021). Sleep disorders are increasingly recognized as major contributors to cerebrovascular disease. Globally, an estimated 30% to 70% of older adults experience sleep-related problems, ranging from insomnia and obstructive sleep apnea (OSA) to circadian rhythm disturbances, and sleep-related movement disorders (Canever et al., 2024). While the traditional focus in stroke prevention has emphasized hypertension, diabetes, atrial fibrillation, and smoking, sleep health represents a missing pillar in vascular risk management—a modifiable and under-recognized factor that warrants equal attention (Bassetti et al., 2020; Gottesman et al., 2024). Indeed, growing evidence suggests a bidirectional relationship: not only do sleep disorders increase the risk of stroke, but stroke itself can disrupt sleep and exacerbate sleep disorders, affecting stroke recovery (Brunetti et al., 2022). In fact, sleep plays a fundamental role in promoting neuroplasticity and brain clearance of metabolic waste, mechanisms that are essential for maintaining brain health and optimizing recovery after stroke. Despite robust evidence, sleep disorders are rarely screened for in routine stroke care. The underdiagnosis of sleep disorders constitutes a missed preventive opportunity and a potential avenue to enhance stroke prevention and rehabilitation (Pincherle et al., 2017).

The eight contributions in this Research Topic reflect the increasing convergence of these fields, highlighting sleep not only as a risk factor and consequence of stroke, but also as a potential window into novel preventive and rehabilitative strategies.

Two studies in this Research Topic examined the role of sleep-disordered breathing (SDB), especially OSA, which has been strongly linked to increased stroke risk and outcome (Baillieul et al., 2022). In a systematic review and meta-analysis, Su et al. found that up to 60% of patients with stroke experience SDB and as many as 45% suffer from moderate-to-severe forms. Importantly, such prevalence has not declined over time. These findings emphasize the critical need for routine SDB screening in stroke care, yet it is not consistently included in standard care pathways for stroke patients. Zhou et al. examined cerebral perfusion in stroke patients with moderate-to-severe OSA. They showed that this population had significantly reduced cerebral blood flow in the acute phase, suggesting that OSA not only predisposes individuals to stroke but may also exacerbate early ischemic damage with extension of infarct volume.

The role of sleep disturbances as cardiovascular risk factor has been analyzed in the study by Wu et al.. The authors assessed the associations between sleep duration, sleep difficulties, and sleep disorders with cardiovascular disease, and also examined the combined impact of these elements as an overall “sleep pattern” variable. Their findings revealed that poor sleep patterns were associated with higher cardiovascular diseases prevalence, particularly among older adults. The study highlights the broader implications of sleep quality as a modifiable factor, relevant for both stroke prevention and overall vascular health.

Stroke recovery is challenging, and specific interventions to improve sleep in order to optimize recovery are scarce. An under investigated yet promising approach that may ameliorate sleep disturbances and enhance stroke recovery is non-invasive brain stimulation (NIBS), which can boost neuroplasticity. In their meta-analysis, Huang et al. examined the effects of different NIBS techniques on post-stroke sleep disorders. Notably, consistent evidence suggests that repetitive transcranial magnetic stimulation (rTMS) can improve sleep quality and architecture, alleviate depressive symptoms, and increase brain-derived neurotrophic factor (BDNF) levels. These promising findings indicate that NIBS warrants further investigation in future clinical trials.

Post-stroke depression (PSD) affects a large proportion of survivors and can hinder recovery. Li et al. explored the role of oxidative stress, proposing that individuals with a higher oxidative balance score (OBS), a measure of oxidative-reductive homeostasis, were less likely to experience PSD. They found that higher OBS was associated with reduced odds of PSD, with a particularly pronounced effect in females. These findings suggest that dietary and lifestyle interventions aimed at reducing the oxidative stress may represent promising avenues to reduce the risk of PSD.

Beyond the stroke population, sleep quality is fundamental for psychological wellbeing (Goldstein and Walker, 2014). Yan et al. tested a behavioral intervention called “happy sensation training” in individuals with insomnia. They observed improvements in sleep architecture and quality, anxiety, and depressive symptoms. Although conducted outside the stroke context, these findings highlight the broader utility of behavioral therapies in post-stroke recovery, especially for improving sleep quality.

Adding further evidence, a neuroimaging study by Zhang et al. revealed significant alterations in resting-state fMRI activity in women with chronic insomnia. They observed widespread alterations across key brain networks, including the default mode network, salience network, central executive network, and limbic system. These disruptions may interfere with emotion regulation and cognitive processing, critical functions for post-stroke adaptation and rehabilitation.

The connection between sleep and cognitive decline has gained increasing attention. In this context, Mayer et al. reviews the growing evidence that links sleep disorders—particularly SDB, insomnia, and abnormal sleep durations—with increased risk of mild cognitive impairment and dementia. While some mechanisms remain under investigation, impaired glymphatic clearance, vascular dysfunction, and neuroinflammation are likely contributors. Importantly, early intervention for sleep disturbances, especially using Continuous Positive Airway Pressure CPAP for OSA, may help preserve cognitive function and delay disease progression. These findings reinforce the potential of sleep health as a modifiable target in dementia prevention strategies and as a pillar for brain health, supporting broader efforts to maintain cognitive resilience in aging populations.

This Research Topic underscores the growing body of evidence supporting the robust association between sleep and cerebrovascular disease. Sleep is a complex and dynamic biological process, influenced by a variety of neural, physiological, and behavioral factors. Given this complexity, a singular therapeutic approach is unlikely to be effective. Instead, the evidence suggests that multicomponent interventions—tailored to individual profiles—may be the most promising strategies for improving outcomes.

Despite this, sleep disorders remain significantly under-recognized and under-addressed in the clinical management of cerebrovascular disorders. The routine evaluation of sleep should be considered a fundamental component of stroke care, both in acute and chronic settings. We hope that this Research Topic will encourage more systematic inclusion of sleep assessments and interventions in both clinical and research frameworks, ultimately advancing the quality of care for individuals affected by stroke.
